# Fish Oil Replacement with Poultry Oil in the Diet of Tiger Puffer (*Takifugu rubripes*): Effects on Growth Performance, Body Composition, and Lipid Metabolism

**DOI:** 10.1155/2022/2337933

**Published:** 2022-09-22

**Authors:** Lin Li, Feiran Zhang, Xiaoxue Meng, Xishuai Cui, Qiang Ma, Yuliang Wei, Mengqing Liang, Houguo Xu

**Affiliations:** ^1^Yellow Sea Fisheries Research Institute, Chinese Academy of Fishery Sciences, 106 Nanjing Road, Qingdao 266071, China; ^2^College of Fisheries and Life Sciences, Shanghai Ocean University, 999 Huchenghuan Road, Shanghai 201306, China; ^3^Laboratory for Marine Fisheries Science and Food Production Processes, Qingdao National Laboratory for Marine Science and Technology, 1 Wenhai Road, Qingdao 266237, China

## Abstract

Booming fish farming results in relative shortage of fish oil (FO), making it urgent to explore alternative lipid sources. This study comprehensively investigated the efficacy of FO replacement with poultry oil (PO) in diets of tiger puffer (average initial body weight, 12.28 g). An 8-week feeding trial was conducted with experimental diets, in which graded levels (0, 25, 50, 75, and 100%, named FO-C, 25PO, 50PO, 75PO, and 100PO, respectively) of FO were replaced with PO. The feeding trial was conducted in a flow-through seawater system. Each diet was fed to triplicate tanks. The results showed that FO replacement with PO did not significantly affect the growth performance of tiger puffer. FO replacement with PO at 50-100% even slightly increased the growth. PO feeding also had marginal effects on fish body composition, except that it increased the liver moisture content. Dietary PO tended to decrease the serum cholesterol and malondialdehyde content but increase the bile acid content. Increasing levels of dietary PO linearly upregulated the hepatic mRNA expression of the cholesterol biosynthesis enzyme, 3-hydroxy-3-methylglutaryl-CoA reductase, whereas high levels of dietary PO significantly upregulated the expression of the critical regulatory enzyme of bile acid biosynthesis, cholesterol 7-alpha-hydroxylase. In conclusion, poultry oil is a good substitution for fish oil in the diets of tiger puffer. Poultry oil could replace 100% added fish oil in the diet of tiger puffer, without adverse effects on growth and body composition.

## 1. Introduction

Fish are the main source of long chain polyunsaturated fatty acids (LC-PUFA) which are beneficial to the health of human consumers [1]. Aquaculture satisfies the growing global demand for fish but also consumes an increasing share of the world's wild fish resources via use of fishmeal and fish oil (FO) in fish feeds [2]. Therefore, increasing levels of alternative sources such as plant ingredients and livestock processing by-products are being used in fish feeds.

Poultry oil (PO) is a by-product of chicken processing, having a relatively low price and a large annual production. Featured with high contents of 16 : 0, 18:1n-9, and 18:2n-6, PO is a potential good lipid source for fish feeds. Partial or complete FO replacement with PO has proved feasible in diets of a series of aquaculture fish species such as Atlantic salmon (*Salmo salar*) [3], rainbow trout (*Oncorhynchus mykiss*) [4], Japanese seabass (*Lateolabrax japonicus*) [5], largemouth bass (*Micropterus salmoides*) [6], yellowtail kingfish (*Seriola lalandi*) [7], barramundi (*Lates calcarifer*) [8], sablefish (*Anoplopoma fimbria*) [9], Florida pompano (*Trachinotus carolinus*) [10], European seabass (*Dicentrarchus labrax*) [11], and gilthead sea bream (*Sparus aurata*) [12]. The saturated and monounsaturated fatty acids (SFA and MUFA, respectively) highly contained in PO were reported to have n-3 LC-PUFA sparing effects. However, the results were not consistent among different species.

The present study was aimed at comprehensively evaluating the efficacy of FO replacement with PO in an important aquaculture fish species, tiger puffer, in terms of growth, body composition, and lipid metabolism. Results of this study will be helpful to the lipid source management in the diets of tiger puffer and will also be inspiring to other farmed fish species.

## 2. Materials and Methods

### 2.1. Experimental Diets

Five isonitrogenous (approximately 46% crude protein), isolipidic (approximately 10% crude lipid), and isoenergetic experimental diets were formulated. FO was used as the sole added oil in the control diet (FO-C). In other diets, FO in the control diet was replaced with PO (refined from duck skin) at different levels, namely, 25%, 50%, 75%, and 100%. The five experimental diets were designated as FO-C, 25PO, 50PO, 75PO, and 100PO, respectively. The formulation and proximate composition of the five experimental diets are presented in Table 1. Fishmeal, soybean meal, corn gluten meal, and brewer's yeast were used as the protein sources, and wheat meal was used as the binder. The diets were made with a laboratory-level single-screw pelleting machine and dried at 55°C [13]. The experimental diets were stored at -20°C prior to use. The fatty acid compositions of oils and diets are presented in Table 2.

### 2.2. Feeding Procedure and Sampling

Tiger puffer juveniles with an average initial body weight of 12.28 g were purchased from Hongqi Modern Fishery Industrial Park (Rizhao, China) and reared in Yellow Sea Aquaculture Co., Ltd. (Yantai, China) for the experimental use. Before the feeding trial, lower teeth of the experimental fish were cut short in order to prevent cannibalism, and the fish were temporarily raised in polyethylene tanks (2 m^3^) with commercial feeds for 7 days to acclimate to the experimental conditions. The feeding trial was conducted in a flow-through seawater system. At the beginning of the experiment, 600 healthy fish were randomly selected and divided into 15 experimental tanks (0.7 × 0.7 × 0.4 m). Each diet was randomly fed to triplicate tanks, and each tank was stocked with 40 fish. Fish were hand-fed to apparent satiation three times daily (7 : 00, 12 : 00, and 18 : 00). The feeding trial lasted 8 weeks. Natural photoperiod was applied throughout the experiment. During the whole feeding trial, the water temperature ranged from 19 to 24°C; salinity, 28-32; pH, 7.6-7.8; dissolved oxygen > 6 mg/L; ammonia-N < 0.5 mg/L; and nitrite < 0.2 mg/L.

At the end of the feeding trial, before sampling, fish were firstly fasted for 24 hours. Fish weight and survival in each tank were measured. After anesthetized with eugenol (1 eugenol: 10,000 water), 3 fish were randomly collected from each tank for the analysis of proximate composition. Four more fish were randomly selected from each tank, and the serum, muscle, liver, and intestine samples were collected. The blood was collected from the caudal vein and kept at room temperature for 2 h and then at 4°C for 6 h. Centrifugation (836 × *g*, 10 min, 4°C) was then conducted, and the straw-colored supernatants were collected as serum samples. From each fish, two pieces of dorsal muscles, two pieces of liver tip tissue, and one piece of midgut (about 1.0 cm) were collected. The samples for Real-Time quantitative Polymerase Chain Reaction (RT-qPCR) studies were immediately frozen with liquid nitrogen and then stored at -76°C before use. The tissue samples for proximate composition analysis were stored at -20°C before use. All sampling protocols, as well as all fish rearing practices, were reviewed and approved by the Animal Care and Use Committee of Yellow Sea Fisheries Research Institute.

### 2.3. Analysis of the Proximate Composition of Fish and Diets

Proximate composition analysis of experimental diets and the whole body, muscle, and liver was performed according to the standard methods of Association of Official Analytical Chemists (AOAC). In brief, the moisture content was measured by drying the samples of diets and fish to a constant weight at 105°C; the protein content was assayed by measuring nitrogen content (*N* × 6.25) using the Kjeldahl method; the lipid content in the diet and whole body was assayed with petroleum ether extraction using the Soxhlet method (but the lipid in muscle and liver was extracted with the chloroform-methanol method), and the ash content was measured by incineration in a muffle furnace at 550°C for 8 h.

### 2.4. Biochemical Parameters of Serum

Serum samples of four fish from each tank were pooled. The concentration of total cholesterol (TC), total triglyceride (TG), total bile acid (TBA), malondialdehyde (MDA), high-density lipoprotein cholesterol (HDL-C), and low-density lipoprotein cholesterol (LDL-C) in serum was measured with commercial kits supplied by Nanjing Jiancheng Bioengineering Institute (Nanjing, China).

### 2.5. RNA Extraction, cDNA Synthesis, and Real-Time Quantitative Polymerase Chain Reaction (RT-qPCR) Analysis

Total RNA in liver samples was extracted with RNAiso Plus (TaKaRa Biotechnology (Dalian) Co., Ltd., Dalian, China). Evo M-MLV RT Mix Kit with gDNA Clean for qPCR (Accurate Biotechnology (Hunan) Co., Ltd., Hunan, China) was used for reverse transcription. SYBR Green Premix Pro Taq HS qPCR Kit II (Accurate Biotechnology (Hunan) Co., Ltd., Hunan, China) and a quantitative thermal cycler (Roche LightCycler 96, Basel, Switzerland) were used for the RT-qPCR. The specific primers for lipid metabolism genes and reference genes are presented in Table 3. The amplification efficiency for all primers was 95~105%, and the coefficients of linear regression were >0.99. The PCR reaction system consists of 1 *μ*L cDNA template, 0.4 *μ*L forward primer (10 *μ*M), 0.4 L reverse primer (10 *μ*M), 5 *μ*L SYBR Green Pro Taq HS Premix II, and 3.2 *μ*L sterilized water. The program was as follows: 95°C for 30 s followed by 40 cycles of “95°C for 5 s, 57°C for 30 s, and 72°C for 30 s.” Melting curve analysis (1.85°C increment/min from 58°C to 95°C) was performed after the amplification phase for validation of a sole product. The mRNA expression was calculated with the 2^−*ΔΔ*Ct^ method [14].

### 2.6. Mitochondrial DNA Copy Number

DNA was extracted from muscle and liver samples with DP324 kit (Tiangen Biochemical Technology Co., Ltd., Beijing, China). Specific primers for 16S rRNA and cytochrome B (CYTB) of mitochondrial DNA were designed (Table 3). *β*-Actin and EF1*Α* were used as the internal references. PCR amplification was performed as previously described in Section 2.5.

### 2.7. Statistical Analyses

All data were analyzed with one-way ANOVA in SPSS 16.0. Prior to analysis, all data were tested for normal distribution using Shapiro-Wilk test, and the homogeneity of variance was tested with Levene's test. Multiple comparisons were performed using Tukey's test, and the significance level was decided when *P* < 0.05. The results were expressed as mean ± standard error.

## 3. Results

### 3.1. Growth Performances, Somatic Indices, and Body Compositions

No significant difference was observed in survival, feed efficiency, and weight gain of fish among different groups (*P* < 0.05, Table 4). However, the weight gain in groups 50PO, 75PO, and 100PO was slightly higher compared to other groups. Group 25PO showed a significantly lower VSI than group FO-C (*P* < 0.05), but no significant difference among groups was observed in other somatic indices.

The PO supplementation had marginal effects on the proximate composition of whole fish body, muscle, and liver (Table 5). Dietary PO supplementation significantly increased the moisture content of the liver (*P* < 0.05).

### 3.2. Serum Biochemical Parameters

In general, the contents of cholesterol including TC, HDL-C, and LDL-C were decreased by the PO supplementation (Table 6). Increasing levels of dietary PO linearly decreased the serum MDA concentration (*P* < 0.05).

### 3.3. Hepatic mRNA Expression of Lipid Metabolism Genes

The dietary PO supplementation had very little influence on the hepatic mRNA expression of most lipid metabolism genes (Table 7). Nevertheless, increasing dietary PO levels linearly upregulated the gene expression of *hmgcr*, and compared to group FO-C, group 100PO showed significantly higher gene expression of *cyp*7*a*1.

### 3.4. Mitochondrial DNA Copy Number

**Figure 1:**
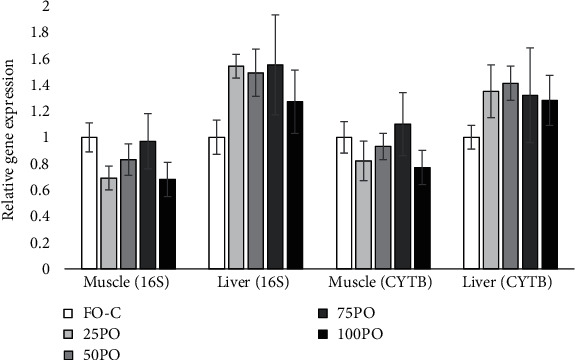
Mitochondrial DNA copy number (relative gene expression of 16S rRNA and cytochrome B in mitochondrial DNA) in the liver and muscle of experimental tiger puffer.

The PO supplementation did not significantly affect the relative gene expression of 16S rRNA and cytochrome B in the mitochondrial DHA of both muscle and liver (Figure 1).

## 4. Discussion

Growth performance is the most valuable indicator of fish nutritional status. The current study revealed that fish oil (FO) replacement with poultry oil (PO) had no significant effects on the weight gain and feed utilization of tiger puffer, indicating the high potential of PO as dietary lipid source. This finding was consistent with previous studies on other fish species such as rainbow trout (2/3 FO replacement) [4], Japanese seabass (50% replacement) [5], yellowtail kingfish (100% replacement) [7], largemouth bass (100% replacement) [6], barramundi (100% replacement) [8], and Florida pompano (75% replacement) [10]. However, other studies showed that the final body weight of gilthead sea bream fed PO was significantly lower than that in the FO control group [12], indicating this species may have a lower capacity to utilize PO.

When different oils were compared in tiger puffer diets, PO resulted in better fish growth than linseed oil, rapeseed oil, and beef tallow, which reduced the fish growth when replacing 100% FO [15]. Although other oils such as tiger puffer liver oil, soybean oil, and palm oil also resulted in comparable growth performance with the FO group, PO resulted in even slightly higher weight gain than the FO control group. The growth-promoting effects of PO could be due to more balanced contents of SFA and MUFA in PO-based diets. These fatty acids, such as 18:1n-9, 16:1n-7, and 16 : 0, are the most preferred substrates for catabolism via *β*-oxidation in fish [16, 17]. It has been demonstrated in many fish studies that a balanced dietary supply of SFA and MUFA limits the metabolic energy required for lipogenesis processes as well as the extent of n-3 LC-PUFA *β*-oxidation, which is called the “n-3 LC-PUFA sparing effect” [18–27]. In this study, the mitochondrial DNA copy number, which is indicative of basic energy supply status, in both muscle and liver of experimental tiger puffer was not significantly different between the FO and PO groups. Moreover, the hepatic expression of lipid metabolism genes was also very marginally affected by the dietary PO. This provided new evidences for the n-3 LC-PUFA sparing effect of SFA and MUFA in PO.

Apart from the growth performance, the somatic indices such as HSI, VSI, and condition factor were mildly affected by dietary PO too. Only the HSI was lowered in group 25PO compared to the FO control. Similar results were found when dietary FO was replaced by linseed oil in diets of tiger puffer [28]. The fish body composition was also very marginally affected by FO replacement with PO, similar to what observed in rainbow trout [4], European seabass [11], and gilthead sea bream [12]. In particular, very little change was observed in the proximate composition of muscle, indicating the little influence of dietary PO on fillet quality. For tiger puffer, the liver is also an edible organ. The liver moisture content of tiger puffer was significantly increased by dietary PO supplementation. Increased moisture content of liver is a favorable change for many consumers, due to the fact that tiger puffer stores lipid predominantly in the liver, leading to an already very high lipid content in the liver. Different from this study, the study on largemouth bass showed that dietary PO inclusion decreased the moisture content of fish liver [6]. This discrepancy may be related to different lipid contents in the liver of different fish species.

Regarding the hematological parameters, dietary inclusion of PO linearly reduced the concentrations of cholesterol and MDA. In general, PO contained lower cholesterol level than FO [29]. The decrease of MDA content by dietary PO could be due to the fact that PO had lower LC-PUFA contents than FO and consequently faced with lower peroxidation stress. Similar results were observed in other tiger puffer studies when linseed oil, soybean oil, rapeseed oil, palm oil, and beef tallow were used to replace FO [15, 28]. Although dietary PO reduced the serum cholesterol content, complete FO replacement with PO increased the content of total bile acid, which is a product of cholesterol metabolism. The gene expression results showed that dietary PO upregulated the hepatic gene expression of both *hmgcr*, which is the rate-limiting enzyme in the synthesis of cholesterol [30, 31], and *cyp*7*a*1, which functions as a critical regulatory enzyme of bile acid biosynthesis [32]. This indicates that dietary PO may stimulate the biosynthesis of cholesterol and bile acid in independent ways, similar to the simulating effects of dietary taurine observed in a recent study on tiger puffer [33]. Nevertheless, it remains unknown in what mechanisms dietary PO stimulated the biosynthesis of bile acid. This warrants further studies.

In conclusion, results of this study suggested that in terms of growth performance, poultry oil is a good potential lipid source in diets of farmed tiger puffer. Fish oil replacement with poultry oil also had marginal effects on the body composition and lipid metabolism of juvenile tiger puffer.

## Figures and Tables

**Table 1:** Formulation and proximate composition of the experimental diets (% dry matter basis).

Ingredients	FO-C	25PO	50PO	75PO	100PO
Fish meal	42	42	42	42	42
Corn gluten meal	8	8	8	8	8
Soybean meal	14	14	14	14	14
Wheat meal	20.68	20.68	20.68	20.68	20.68
Brewer's yeast	5	5	5	5	5
Mineral premix^a^	0.5	0.5	0.5	0.5	0.5
Vitamin premix^a^	1	1	1	1	1
Monocalcium phosphate	1	1	1	1	1
L-Ascorbyl-2-polyphosphate	0.2	0.2	0.2	0.2	0.2
Choline chloride	0.2	0.2	0.2	0.2	0.2
Betaine	0.3	0.3	0.3	0.3	0.3
Ethoxyquin	0.02	0.02	0.02	0.02	0.02
Mold inhibitor^b^	0.1	0.1	0.1	0.1	0.1
Soya lecithin	1	1	1	1	1
Fish oil	6	4.5	3	1.5	0
Poultry oil^c^	0	1.5	3	4.5	6
Proximate composition					
Crude protein	45.40	45.84	45.65	46.26	46.23
Crude lipid	9.31	10.06	9.99	10.08	9.89
Ash	9.41	9.47	9.56	9.56	9.47

^a^Mineral premix and vitamin premix, designed for marine fish, were purchased from Qingdao Master Biotech Co., Ltd., Qingdao, China. ^b^Contained 50% calcium propionic acid and 50% fumaric acid. ^c^Poultry oil was purchased from Shandong Haiding Agriculture and Animal Husbandry Co., Ltd., Shandong, China.

**Table 2:** Fatty acid composition of fish oil, poultry oil, and experimental diets (%TFA).

Fatty acid	Oil		Diet				
	Fish oil	Poultry oil	FO-C	25PO	50PO	75PO	100PO
14 : 0	5.33	0.58	5.55	4.76	4.06	3.35	2.69
16 : 0	18.60	26.61	21.24	22.32	22.99	23.64	24.99
18 : 0	4.58	5.60	4.58	4.76	4.79	4.76	4.94
∑SFA	28.51	32.79	31.37	31.84	31.84	31.75	32.62
16:1n-7	5.34	2.91	6.17	5.74	5.15	4.75	4.60
18:1n-9	16.12	44.30	15.12	18.76	22.11	25.51	29.73
20:1n-9	1.48	0.42	0.97	0.85	0.71	0.57	0.46
∑MUFA	22.94	47.63	22.25	25.35	27.97	30.84	34.80
18:2n-6	12.28	15.11	13.25	14.20	14.41	14.63	15.11
20:2n-6	0.22	0.12	0.22	0.22	0.19	0.18	0.17
20:4n-6	0.55	0.21	0.68	0.69	0.60	0.58	0.52
22:2n-6	0.38	ND	0.33	0.28	0.24	0.17	0.12
∑n-6PUFA	13.43	15.44	14.48	15.38	15.45	15.56	15.92
18:3n-3	1.69	0.69	1.52	1.38	1.28	1.12	1.03
20:5n-3	8.15	0.06	9.66	8.38	7.24	6.07	5.11
22:5n-3	0.82	0.03	1.17	1.05	0.98	0.85	0.77
22:6n-3	8.97	0.02	7.36	6.17	5.24	4.02	2.95
∑n-3PUFA	19.63	0.80	19.70	16.98	14.74	12.06	9.87
∑n-3/∑n-6	1.46	0.05	1.36	1.10	0.95	0.77	0.62

TFA: total fatty acid; SFA: saturated fatty acid; MUFA: mono-unsaturated fatty acid; n-6 PUFA: n-6 poly-unsaturated fatty acid; n-3 PUFA: n-3 poly-unsaturated fatty acid; ND: nondetectable.

**Table 3:** Sequence information of the primers used in this work.

Primer	Sequence (5′-3′)	GenBank reference	PL (bp)
Lipid metabolism genes			
Lipogenesis			
*acacβ*-F	GAAAGGTTTGCTGTGCGACTA	XM_011615767.1	154
*acacβ*-R	TTACATCAGCGACCATTTCAGT		
*fas*-F	CTTTGCCGCTGTCATTCG	XM_011619859.1	78
*fas*-R	TGTCTCAACCCATTTGTAGTCG		
*β*-Oxidation			
*cpt-1*-F	GGGGTTTGTGGTCAAGTTAGG	XM_011607269.1	186
*cpt-1*-R	ATAGATCCGTGGCGCTCAT		
*vlc*-qF	CGCTGTTCTTGGTGTTGGAC	XM_003969871.3	276
*vlc*-qR	GAGATTTGCTGCGGATGTTG		
*acox1*-qF	GCACGGCATCGCAAGTAAC	XM_029850253.1	145
*acox1*-qR	GAGATCGAAGGCATCCACC		
*acox3*-qF	GACTGTGGCTATCCGCTTCT	XM_029839734.1	214
*acox3*-qR	TTCCTGTCGGTCACTCTTGT		
*ehhadh*-qF	GGCACAATGGGAAGAGGCATT	XM_003961946.3	185
*ehhadh*-qR	TGGACGGTTTCGCTGTAGGTA		
*acaa1*-qF	GGACAACAGCAAAGCAAGAG	XM_029849183.1	110
*acaa1*-qR	ACCAGAAAAAACAGCCAAAA		
*acaa2*-qF	ACGGGGGTGTTTTGAAGGA	XM_003975006.3	159
*acaa2*-qR	CATGACGGGCAATGTAGGG		
*gpat*-F	CCCGTTCACAAATCCCACA	XM_011621885.1	235
*gpat*-R	GGCACAACAACTCCTCCGTAT		
*dgat1*-F	TGGTTTGTGAGCCGTTTCC	XM_003969352.2	185
*dgat1*-R	CTGGCATTCGTTTGACTTCG		
*mgat2a*-F	AAAGGCTTCATTAAATTGGC	XM_003978609.3	223
*mgat2a*-R	TGATGGCTTGTCTGTAGGG		
Hydrolysis of glycerides			
*atgl-F*	CCAACCTCTACAGGGTCTCA	XM_003967696.3	119
*atgl-R*	GTTTAGCAGCCCGTTCTTC		
*daglα*-F	CTGTTGGTGGAGTTGGTGTATG	XM_011610175.1	72
*daglα*-R	ATCAGAGCACGGCTGGTAAT		
*hsl*-F	CTCTTGCTATCGGTCTTGTGG	XM_011621066.1	113
*hsl*-R	TTCTGGGTCAATGGCATACTT		
*mgll*-F	CCATCCAGTCAAAGTGGGTCT	XM_003963030.2	110
*mgll*-R	CATCAGCTGCATGCCGAA		
Lipid digestion			
*bsal*-F	TTGAAGATGACTGACCCCGA	XM_003978375.2	162
*bsal*-R	GATGTCTGCTGCGTTGTGAA		
*lp*-F	CGTTTTCTCCTGTTCACCC	XM_029832009.1	97
*lp*-R	GACTCGTCCTCATCCCACT		
Lipid transport			
*lpl*-F	AGGGTCCACATCCGCAAA	NM_001305600.1	157
*lpl*-R	GTTTCTCCTTGCGGCTCAT		
*lipc*-F	GCGGCTTCAACAGCAGTAA	XM_011610357.1	215
*lipc*-R	GAGGTGCGCTATGTCTTTCC		
*fabp1*-F	CCATCGGTCTCCCTGATGAAG	XM_003974807.3	121
*fabp1*-R	TTGACCGTTACCTTCGGTCC		
*fabp10a*-F	CTGTGACCAACTCCTTTACCAT	XM_003965635.3	150
*fabp10a*-R	TCTCTCCACCTTTGAGCTCCTG		
*fatp1*-F	ATTGCAGACACCACAGGGAG	XM_003964742.3	219
*fatp1*-R	ATATCGTGACGCTCGTGCAT		
*apoa1*-F	CGATGACGCCGAGTACAAA	AB183289.1	104
*apoa1*-R	CGGTTATGGGAGAAACGCTA		
*apoa4*-F	TGCTTTCTGGGACTATGTTGC	NM_001078591.1	124
*apoa4*-R	GTTGACTTTGTCGGCACTCTC		
*apob100*-F	AGGGACATAGTCAAACCAAGGA	XM_011619944.1	127
*apob100*-R	AGAACACGAAGGCTGGACAC		
*apoe1*-F	TATTCAGACCCGCACCTCA	NM_001078592.1	201
*apoe1*-R	ATTTCCTCCATCTTGTCCTCC		
*mttp*-F	ATGCTAAGGGTCTGGTTCTGC	XM_011612378.1	124
*mttp*-R	ATGTCAGTGCTGCCGATCTT		
Lipid metabolism-related transcriptional factors			
*srebf1*-F	TTTCAGCATCCCACCTTCC	XM_011603881.1	158
*srebf1*-R	GGTGAACCGTGAGGACAACTA		
*pparα1*-F	TCAGTAGTTTATGGGTTGGTGG	NM_001097630.1	119
*pparα1*-R	GCGTGGACTCCGTAGTGGTA		
*pparα2*-F	CCAGAAGAAGAACCGCAACA	NM_001097629.1	149
*pparα2*-R	CCTCTTTCTCCACCATCTTGTT		
*pparβ*-F	AGCTGGAATACGACCGATGT	AB275887.1	249
*pparβ*-R	TCTTCAGGTAGGCGGAGTTG		
*pparγ*-F	CGCTGTCCCGACATCTGTAT	NM_001097627.1	146
*pparγ*-R	GAACTGCTCGCCTTCCATT		
*fxr*-F	GTGAACGACCACAAGTTTACCC	XM_003967283.2	166
*fxr*-R	AGACCAACAGATTACACCGGAT		
*lxrα*-F	GTGACGCACCACTAACAGCA	XM_011609917.1	191
*lxrα*-R	CTGACAACACCGAGCAAGACT		
*hnf4α*-F	GAGCCACGGGCAAACACTA	XM_011619034.1	199
*hnf4α*-R	AGGGTCCTACCTTCTTTCTTCAT		
*lrh-1*-F	CGCTGACATGCTGCCTAAA	XM_003974281.2	140
*lrh-1*-R	TCTCGTCCAAGTCTTCGTCAT		
Cholesterol and bile acid biosynthesis			
*hmgcr*-F	GCTGCTGGCAATCAAGTACAT	XM_003974466.2	237
*hmgcr*-R	AAACATACAACTCCTTCCTACAGC		
*cyp7a1*-F	CCTACCTGCTACCTTCTGGAGT	XM_003975521.2	143
*cyp7a1*-R	TCCTCTTTGGCAACACGAA		
Reference gene			
*β-Actin*-F	GAGAGGGAAATCGTGCGTGA	XM_003964421.3	186
*β-Actin*-R	GAAGGATGGCTGGAAGAGGG		
*ef1α*-F	TTGGAGGCATTGGAACTGT	NM_001037873.1	86
*ef1α*-R	GTTGACGGGAGCAAAGGT		
Mitochondrial DNA			
16S rRNA-F	ATGTGGACCTGTATGAATGGC	NC_004299.1	119
16S rRNA-R	CTCCATAGGGTCTTCTCGTCTT		
CYTB-F	CCTCCTGGGCTTCACAATCA	NC_004299.1	123
CYTB-R	TTAATGTGGGCGGGGGTAAC		
*β*-Actin-F	GACGCAAAACCTCCGAACTG	Gene ID 101079312	129
*β*-Actin-R	CCTCCAAACGGATCAGCACA		
EF1*Α*-F	TGGCCTTTAGCCGAATGAGG	Gene ID 653026	117
EF1*Α*-R	TGTCGGGCCAATCAATCCAG		

*acacβ*: acetyl-CoA carboxylase beta; *fas*: fatty acid synthase; *cpt-1*: carnitine O-palmitoyltransferase-1; *vlcs*: very long-chain acyl-CoA synthetase; *acox1*: acyl-CoA oxidase 1, palmitoyl; *acox3*: acyl-CoA oxidase 3, pristanoyl; *ehhadh*: enoyl-CoA hydratase and 3-hydroxyacyl CoA dehydrogenase; *acaa*: acetyl-CoA acyltransferase; *gpat*: glycerol-3-phosphate acyltransferase; *dgat1*: diacylglycerol O-acyltransferase 1; *mgat2a*: 2-acylglycerol O-acyltransferase 2-A-like (LOC101069338); *atgl*: adipose triglyceride lipase (patatin-like phospholipase domain containing 2 (pnpla2)); *daglα*: diacylglycerol lipase, alpha; *hsl*: hormone-sensitive lipase; *mgll*: monoglyceride lipase; *bsal*: bile acid activated lipase; *lp*: inactive pancreatic lipase-related protein 1-like (LOC101064949); *lpl*: lipoprotein lipase; *lipc*: lipase, hepatic; *fabp*: fatty acid binding protein; *fatp*: fatty acid transport protein (solute carrier family 27 member 1 (slc27a1)); *apo*: apolipoprotein; *mttp*: microsomal triglyceride transfer protein; *srebf1*: sterol regulatory element binding transcription factor 1; *ppar*: peroxisome proliferator-activated receptor; *fxr*: farnesoid X receptor (nuclear receptor subfamily 1, group H, member 4, NR1H4); *lxrα*: liver X receptor alpha (nuclear receptor subfamily 1, group H, member 3, NR1H3); *hnf4α*: hepatocyte nuclear factor 4, alpha; *lrh-1*: liver receptor homolog-1 (nuclear receptor subfamily 5, group A, member 2, NR5A2); *hmgcr*: 3-hydroxy-3-methylglutaryl-CoA reductase; *cyp*7*a*1: cholesterol 7-alpha-hydroxylase (cytochrome P450 family 7 subfamily A member 1); CYTB: cytochrome B; PL: product length.

**Table 4:** Growth performances and somatic parameters of experimental tiger puffer (mean ± standard error).

Parameters	FO-C	25PO	50PO	75PO	100PO
IBW (g)	12.28 ± 0.00	12.28 ± 0.01	12.28 ± 0.00	12.28 ± 0.01	12.27 ± 0.01
FBW (g)	39.69 ± 1.57	40.28 ± 0.93	44.26 ± 0.98	42.58 ± 2.05	43.14 ± 2.64
WG (%)	223.4 ± 12.79	228.2 ± 7.51	260.6 ± 8.01	246.7 ± 16.68	251.6 ± 21.33
FI (%)	1.76 ± 0.04^ab^	1.81 ± 0.03^b^	1.68 ± 0.03^a^	1.67 ± 0.03^a^	1.68 ± 0.03^a^
FER	0.95 ± 0.03	0.92 ± 0.02	1.00 ± 0.05	0.97 ± 0.03	1.03 ± 0.02
Survival (%)	70.00 ± 3.82	67.5 ± 0.00	60.00 ± 5.20	58.33 ± 5.07	65.00 ± 1.44
HSI (%)	8.75 ± 0.16	7.99 ± 0.46	8.45 ± 0.26	8.88 ± 0.35	8.43 ± 0.16
VSI (%)	14.84 ± 0.27^b^	13.28 ± 0.41^a^	14.05 ± 0.61^ab^	14.53 ± 0.62^ab^	13.92 ± 0.13^ab^
CF (g/cm^3^)	3.23 ± 0.13	3.19 ± 0.09	3.14 ± 0.20	3.16 ± 0.15	3.27 ± 0.19

Data in thea same row not sharing a the same superscript letter are significantly different (*P* < 0.05). IBW: initial body weight; FBW: final body weight; WG(weight gain) = (FBW − IBW)/IBW × 100; FI(feed intake) = feed dry weight/[experimental days × (IBW + FBW)/2] × 100; FER(feed efficiency ratio) = weight gain/feed intake × 100; Survival = final fish number/initial fish number × 100.; HSI(hepatosomatic index) = (liver weight/body weight) × 100; VSI(viserasomatic index) = (viscera weight/body weight) × 100; CF(condition factor) = weight of fish/length of fish 3 × 100.

**Table 5:** Proximate composition of whole body, muscle, and liver of experimental tiger puffer (% wet weight, mean ± standard error).

Parameters	FO-C	25PO	50PO	75PO	100PO
Whole body					
Moisture (%)	77.68 ± 0.05	77.62 ± 0.54	77.64 ± 0.09	77.32 ± 0.07	77.90 ± 0.13
Crude protein (% w.w.)	14.97 ± 0.15	14.97 ± 0.33	14.88 ± 0.28	15.04 ± 0.10	14.89 ± 0.21
Crude lipid (% w.w.)	4.60 ± 0.21	4.47 ± 0.37	4.40 ± 0.20	4.91 ± 0.16	4.18 ± 0.20
Muscle					
Moisture (%)	79.03 ± 0.10	79.19 ± 0.32	78.77 ± 0.03	78.96 ± 0.12	78.66 ± 0.30
Crude protein (% w.w.)	18.16 ± 0.15^ab^	17.92 ± 0.12^a^	18.42 ± 0.09^b^	18.15 ± 0.07^ab^	18.34 ± 0.16^b^
Crude lipid (% w.w.)	0.71 ± 0.02^ab^	0.68 ± 0.01^a^	0.76 ± 0.02^b^	0.72 ± 0.03^ab^	0.74 ± 0.02^ab^
Liver					
Moisture (%)	25.15 ± 0.71^a^	31.5 ± 0.86^b^	30.48 ± 0.47^b^	30.13 ± 1.06^b^	32.28 ± 1.82^b^
Crude lipid (% w.w.)	49.78 ± 0.33	49.65 ± 2.30	48.23 ± 0.89	51.37 ± 1.80	48.85 ± 3.05

Data in the same row not sharing the same superscript letter are significantly different (*P* < 0.05). w.w.: wet weight.

**Table 6:** Serum biochemical indices of experimental tiger puffer (mean ± standard error).

Parameters	FO-C	25PO	50PO	75PO	100PO
TG (mmol L^−1^)	1.51 ± 0.05^ab^	1.70 ± 0.11^ab^	1.86 ± 0.12^b^	1.75 ± 0.10^ab^	1.40 ± 0.05^a^
TC (mmol L^−1^)	6.01 ± 0.15^b^	5.61 ± 0.17^ab^	5.34 ± 0.15^ab^	5.62 ± 0.09^ab^	5.12 ± 0.22^a^
HDL-C (mmol L^−1^)	2.63 ± 0.11	2.28 ± 0.43	2.56 ± 0.30	2.14 ± 0.04	1.69 ± 0.15
LDL-C (mmol L^−1^)	3.39 ± 0.20^b^	3.03 ± 0.37^b^	2.47 ± 0.11^ab^	2.49 ± 0.02^ab^	2.09 ± 0.11^a^
TBA (*μ*mol L^−1^)	0.78 ± 0.11^ab^	0.76 ± 0.06^a^	0.77 ± 0.03^a^	0.87 ± 0.08^ab^	1.13 ± 0.05^b^
MDA (nmol ml^−1^)	11.10 ± 0.69^c^	10.78 ± 0.75^bc^	8.69 ± 0.11^ab^	8.59 ± 0.13^ab^	6.56 ± 0.41^a^

Data in the same row not sharing the same superscript letter are significantly different (*P* < 0.05). TG: triacylglycerol; TC: total cholesterol; HDL-C: high-density lipoprotein cholesterol; LDL-C: low-density lipoprotein cholesterol; TBA: total bile acid; MDA: malondialdehyde.

**Table 7:** Relative mRNA expression levels of genes related to lipid metabolism in the liver of experimental tiger puffer at the end of the growing-out period (mean ± standard error).

Gene	FO-C	25PO	50PO	75PO	100PO
Lipogenesis					
*acacβ*	1.00 ± 0.12	1.16 ± 0.10	1.20 ± 0.12	1.09 ± 0.25	1.62 ± 0.11
*fas*	1.00 ± 0.06	1.66 ± 0.81	2.21 ± 0.43	1.91 ± 0.25	1.84 ± 0.23
*β*-Oxidation					
*cpt-*1	1.00 ± 0.06	1.11 ± 0.19	0.98 ± 0.05	0.83 ± 0.18	1.19 ± 0.26
*vlcs*	1.00 ± 0.14	1.93 ± 0.63	2.25 ± 0.45	1.67 ± 0.25	2.23 ± 0.47
*acox*1	1.00 ± 0.03	1.45 ± 0.29	1.33 ± 0.24	1.32 ± 0.26	1.75 ± 0.24
*acox*3	1.00 ± 0.07^ab^	1.29 ± 0.05^ab^	1.45 ± 0.10^b^	0.97 ± 0.04^a^	1.40 ± 0.14^ab^
*ehhadh*	1.00 ± 0.04	1.63 ± 0.06	1.37 ± 0.13	1.12 ± 0.06	1.58 ± 0.19
*acaa*1	1.00 ± 0.05^a^	1.49 ± 0.09^b^	1.26 ± 0.14^ab^	0.89 ± 0.06^a^	1.46 ± 0.05^b^
*acaa*2	1.00 ± 0.12	1.05 ± 0.21	1.27 ± 0.39	0.64 ± 0.08	1.08 ± 0.10
Biosynthesis of glycerides					
*gpat*	1.00 ± 0.07	1.11 ± 0.17	1.12 ± 0.21	1.49 ± 0.57	1.00 ± 0.04
*dgat*1	1.00 ± 0.18	1.22 ± 0.43	1.53 ± 0.11	1.48 ± 0.22	1.88 ± 0.39
*mgat*2*a*	1.00 ± 0.13^a^	0.92 ± 0.17^ab^	0.84 ± 0.05^ab^	0.48 ± 0.04^b^	0.81 ± 0.08^ab^
Hydrolysis of glycerides					
*atgl*	1.00 ± 0.17	0.99 ± 0.37	0.70 ± 0.23	0.69 ± 0.27	1.10 ± 0.25
*daglα*	1.00 ± 0.12	1.12 ± 0.05	1.03 ± 0.05	0.88 ± 0.15	1.18 ± 0.13
*hsl*	1.00 ± 0.26	0.93 ± 0.17	0.74 ± 0.17	1.07 ± 0.33	1.07 ± 0.03
*mgll*	1.00 ± 0.12^a^	1.62 ± 0.58^ab^	1.84 ± 0.17^ab^	1.54 ± 0.45^ab^	2.72 ± 0.05^b^
Lipid digestion					
*bsal*	1.00 ± 0.14	0.74 ± 0.15	0.81 ± 0.17	0.69 ± 0.13	1.11 ± 0.03
*lp*	1.00 ± 0.10	1.31 ± 0.17	1.20 ± 0.41	1.75 ± 0.94	1.39 ± 0.37
Lipid transport					
*lpl*	1.00 ± 0.13	1.11 ± 0.04	0.92 ± 0.14	0.91 ± 0.13	1.39 ± 0.10
*lipc*	1.00 ± 0.12	0.79 ± 0.20	0.78 ± 0.13	0.76 ± 0.12	0.93 ± 0.19
*fabp1*	1.00 ± 0.25	1.33 ± 0.39	1.63 ± 0.42	1.11 ± 0.47	1.94 ± 0.36
*fabp10a*	1.00 ± 0.22	0.73 ± 0.02	0.89 ± 0.07	0.99 ± 0.37	1.04 ± 0.28
*fatp1*	1.00 ± 0.08	1.41 ± 0.62	1.02 ± 0.01	0.79 ± 0.03	1.13 ± 0.11
*apoa*1	1.00 ± 0.11	0.80 ± 0.21	1.05 ± 0.03	0.79 ± 0.05	1.06 ± 0.17
*apoa*4	1.00 ± 0.08	1.01 ± 0.14	0.84 ± 0.22	0.88 ± 0.08	0.89 ± 0.15
*apob*100	1.00 ± 0.03	1.18 ± 0.20	1.22 ± 0.15	1.01 ± 0.19	1.40 ± 0.18
*apoe*1	1.00 ± 0.05	0.93 ± 0.31	0.72 ± 0.06	1.23 ± 0.31	1.03 ± 0.08
*mttp*	1.00 ± 0.01	1.20 ± 0.25	1.28 ± 0.10	1.23 ± 0.17	1.52 ± 0.21
Lipid metabolism-related transcriptional factors					
*srebf*1	1.00 ± 0.06	1.25 ± 0.27	1.22 ± 0.25	1.14 ± 0.19	1.30 ± 0.09
*pparα*1	1.00 ± 0.03	0.93 ± 0.05	1.27 ± 0.23	1.17 ± 0.24	1.37 ± 0.39
*pparα*2	1.00 ± 0.05	1.18 ± 0.03	1.18 ± 0.19	1.01 ± 0.09	1.18 ± 0.22
*pparβ*	1.00 ± 0.14	1.55 ± 0.01	1.25 ± 0.20	1.29 ± 0.27	1.52 ± 0.32
*ppacγ*	1.00 ± 0.03	1.17 ± 0.09	0.97 ± 0.18	0.94 ± 0.05	1.29 ± 0.13
*fxr*	1.00 ± 0.05	0.79 ± 0.18	0.98 ± 0.03	0.78 ± 0.11	1.19 ± 0.25
*lxrα*	1.00 ± 0.12	1.46 ± 0.13	1.24 ± 0.10	1.15 ± 0.19	1.55 ± 0.06
*hnf4α*	1.00 ± 0.03	1.22 ± 0.07	1.16 ± 0.13	0.96 ± 0.25	1.07 ± 0.12
*lrh-*1	1.00 ± 0.06	1.19 ± 0.01	1.08 ± 0.10	1.00 ± 0.07	1.15 ± 0.05
Cholesterol and bile acid biosynthesis					
*hmgcr*	1.00 ± 0.09^a^	1.87 ± 0.21^ab^	2.07 ± 0.46^ab^	2.14 ± 0.46^ab^	3.17 ± 0.63^b^
*cyp*7*a*1	1.00 ± 0.34^a^	1.39 ± 0.61^ab^	1.22 ± 0.14^a^	1.12 ± 0.09^a^	2.88 ± 0.45^b^

Data in the same row not sharing the same superscript letter are significantly different (*P* < 0.05).

## Data Availability

Raw data supporting the conclusions of this manuscript will be made available by the authors, without undue reservation, to any qualified researcher.
